# Formability Limits in Square Tubes and L-Section Profiles

**DOI:** 10.3390/ma18122852

**Published:** 2025-06-17

**Authors:** Inês M. Almeida, João P. G. Magrinho, Maria Beatriz Silva

**Affiliations:** IDMEC, Instituto Superior Técnico, Universidade de Lisboa, Av. Rovisco Pais, 1049-001 Lisboa, Portugal; ines.matias.almeida@tecnico.ulisboa.pt (I.M.A.); joao.magrinho@tecnico.ulisboa.pt (J.P.G.M.)

**Keywords:** thin-walled tubes, L-profiles, formability limits, necking, fracture

## Abstract

Understanding the formability limits of thin-walled tubes with square cross-sections and L-section profiles is crucial for improving manufacturing efficiency and ensuring structural reliability in industries such as automotive and aerospace. Unlike the usually studied circular tubes, square tubes and L-section profiles geometries present unique deformation and fracture behaviours that require specific analysis. To address this gap, this research establishes a novel methodology combining digital image correlation (DIC) with a time-dependent approach and precise thickness measurements, enabling accurate strain measurements essential to the onset of necking and fracture strain identification. Two experimental tests under different forming conditions allowed capturing a distinct range of strain paths leading to failure. This approach allowed the determination of the forming limit points associated with necking and the fracture forming lines associated with crack opening by tension (mode I) and by in-plane shear (mode II). The findings highlight the strong influence of geometry on the fracture mechanisms and provide valuable data for optimizing tube-forming processes for square tubes and L-section profiles, ultimately enhancing the design and performance of lightweight structural components.

## 1. Introduction

Thin-walled structures combine low weight with high strength and resistance to axial, bending, and torsional forces, making them highly versatile across various industries [1]. Available in diverse cross-sectional shapes, including circular and square tubes and profiles, they can be tailored to specific performance requirements. This adaptability is particularly valuable in transportation, where weight reduction, energy efficiency, and durability are key priorities [2]. While bending [3] and end-forming [4] of tubes remain the dominant manufacturing methods, advanced techniques like tube hydroforming [5] have gained traction in mass-producing lightweight components. Additionally, emerging processes such as incremental tube forming [6], friction-spinning [7], and tube narrowing and tube expansion processes [8] present promising alternatives, offering reduced energy consumption and material waste [9].

The growing demand for these structures highlights the need for a comprehensive understanding of how and why failure occurs. This is usually evaluated by two main approaches: defining the process window or establishing the formability limits diagram. The first approach refers to identifying the range of parameters or conditions under which a manufacturing process can operate effectively—commonly known as the process window. An example of this approach was provided by Korkolis and Kyriakides [10], who defined a process window for free tube hydroforming, where Al-6260-T4 tubes were fixed only at their ends. In their study, the process window was characterized by the axial force applied to seal the tube and the internal pressure applied during forming.

The second approach, the formability limit diagram (FLD), is a graphical tool commonly used in sheet metal forming to assess material formability. It defines the strain combinations beyond which the material is likely to fail during forming. An example of this approach was presented by Magrinho et al. [9], who applied different formability tests to thin-walled tubes with circular cross-sections to construct the formability limits by necking and by fracture and validated this methodology for the tube inversion process, confirming that is constrained by local buckling and fracture and thereby validating the previously determined formability limits.

In recent years, the fracture behaviour of circular thin-walled tubes has been of increasing interest. Several experimental studies involving tube bulging tests [11,12] and tube hydroforming tests [13,14] have been conducted to determine the fracture forming limits of thin-walled circular tubes. However, these studies did not address the crack opening mode associated with fracture or necking, nor did they explore the corresponding formability limits in tubular structures with non-circular cross-sections.

In the sheet metal realm, Embury and Duncan [15] originally proposed that the fracture forming limit (FFL) was due to crack opening by tension (mode I of fracture mechanics [16]). Later, Isik et al. [17] stated that the shear fracture forming limit line (SFFL) originates from cracks opened by in-plane shear stresses (mode II of fracture).

Regarding the tube-forming realm, recently the authors have developed experimental methodologies to assess the formability limits of thin-walled tubes by adapting the techniques commonly used in sheet metal forming. Initially, tube expansion with rigid dies was proposed as a way to determine fracture strains and critical ductile damage values [18]. This approach was later refined by introducing tube expansion with elastomers, which allowed for testing under a broader range of conditions, from pure tension to biaxial stretching [9]. As a result, for the first time, the formability limits of thin-walled circular tubes were successfully identified in terms of both necking and fracture.

Later, Magrinho et al. [19] and Suntaxi et al. [20] developed a methodology to determine failure limits due to fracture and necking. By integrating Digital Image Correlation (DIC), thickness measurements, and time-dependent approaches, they achieved a complete characterization of the Fracture Forming Limit (FFL), the Safe Forming Failure Limit (SFFL), and the transition region between them.

This study presents, for the first time, the determination of formability limits by necking and fracture for thin-walled L-section profiles and square cross-section tubes, addressing a gap in the tube-forming literature, where methodologies for such structures remain unexplored. Individual wall specimens extracted from these profiles were tested to isolate and characterize their fundamental deformation behaviour under controlled conditions. This foundational approach eliminates geometric complexities and provides essential baseline data that has not previously been available for these profile types. The results are expected to serve as a critical reference for future investigations involving the full structural response of these components under more complex loading conditions, such as buckling and expansion. These findings not only fill a significant gap in the literature but also contribute to advancing material forming techniques for complex-profiled structural components.

## 2. Materials and Methods

### 2.1. Material Characterization

The experimental work was carried out on commercial AA6063-T6 aluminum tubes with a square cross-section, hereafter called ‘square tubes’, and profiles with an L-section, hereafter called ‘profiles’, for writing simplicity. The dimensions of these geometries are presented in Table 1.

**Figure 1:**
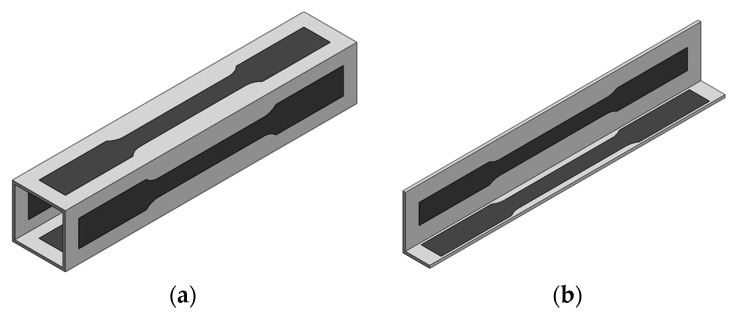
Tensile specimens retrieved from (**a**) the square tube and from (**b**) the profile.

The mechanical properties of the material in both geometries were determined by means of tensile test specimens prepared according to the ASTM E8/E8M-22 standard [21]. The tests were performed using a universal testing machine, Instron 5900R, at room temperature, and the specimens were extracted from the geometries, as shown in Figure 1, through wire electrical discharge machining, with several specimens extracted from each wall of the geometries. The properties determined are summarized in Table 2.

In Table 2, $\sigma_{0.2\%}$ stands for the 0.2% offset yield stress; $\sigma_{UTS}$ is the ultimate tensile strength; A is the elongation at fracture; and r is the anisotropy coefficient. The true stress–strain curves were approximated by the Ludwik–Hollomon law of the material ($\sigma =K.\varepsilon^{n}$), which is characterized by the values of K, which depend on the material and testing conditions, and n, which corresponds to the strain hardening exponent.

Both geometries, despite being made from the same material, were manufactured in different batches using porthole extrusion. This process leads to variations in property values across the walls of each geometry and differences in material properties between the two geometries. Despite this, the differences between the walls of the same geometry were not significant, and therefore the average value was considered.

The anisotropy coefficient, r, was determined as the ratio between the strain in the width and the strain in the thickness, as shown in Equation (1).

(1) $$r=\frac{\varepsilon_{w}}{\varepsilon_{t}}$$

Given the determined values for both geometries, which are significantly below unity, the material is expected to exhibit greater deformation in the thickness direction, with this effect being more pronounced in the profile.

### 2.2. Methodology

The proposed methodology to determine the formability limits by necking and fracture in the square tubes and in the profile aims to extend those already utilized for other geometries. The tests performed were tensile and double-notched shear tests, following the ASTM E8/E8M-22 [21] and ASTM B831-19 [22] standards, respectively, at room temperature and in random order, and at least three tests were performed for each testing condition to ensure reproducibility of the results.

**Figure 2:**
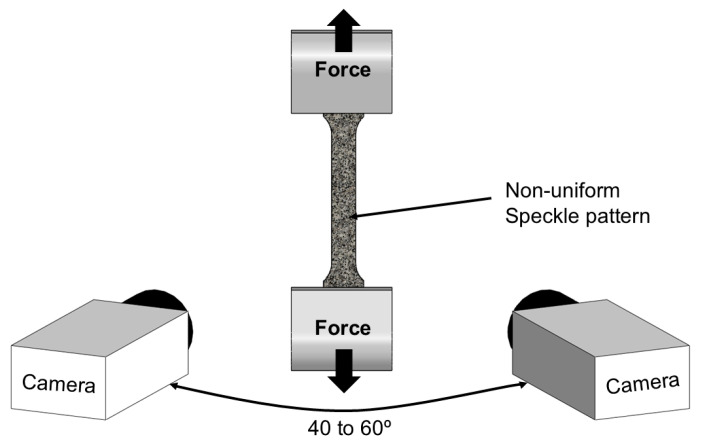
Schematic representation of the experimental setup.

To capture field variables, a Digital Image Correlation (DIC) system from Dantec Dynamics (Skovlunde, Denmark, model Q-400 3D) was used. The specimen surfaces were prepared with a stochastic black speckle pattern on a uniformly painted matte white background to enhance image tracking (see Figure 2). A single spotlight was used for illumination, ensuring a broader measurement region.

The DIC system was equipped with two cameras with 6-megapixel resolution and a 50.2 mm focal length lens with an aperture of f/8 and captured images at a frequency of 10 frames per second. The correlation algorithm was executed using the INSTRA 4D software V4.9.

#### 2.2.1. Formability Limits by Necking

Using the described setup to measure the strains in the specimen during the tensile test, it was possible to determine the onset of necking and the respective critical strains using the time-dependent methodology proposed by Martínez-Donaire et al. [23] in both geometries.

**Figure 3:**
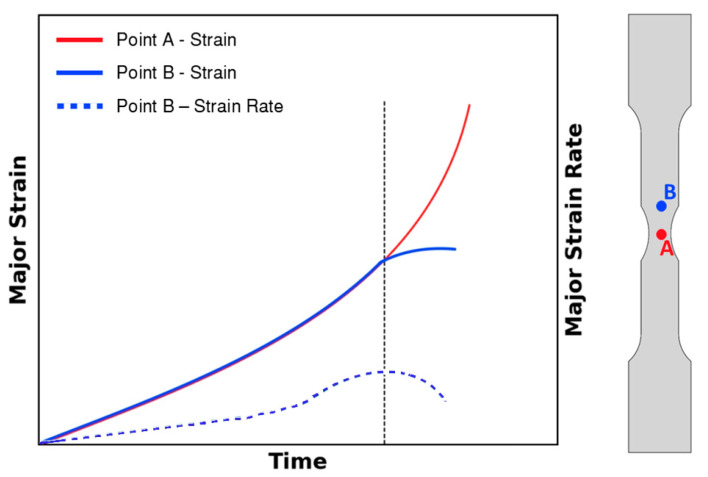
Schematic representation of the time-dependent methodology (adapted from [19]).

To determine the onset of necking the time-dependent methodology requires the identification of two points: one in the specimen’s fracture location and another in the vicinity of the necking area. After identifying these points following the steps described by Martínez-Donaire et al. [23], the onset of necking is determined to be the instant where the maximum value of the major strain rate $\left(\dot{\varepsilon_{1}}\right)$ of the point in the vicinity of the necking area (point B) is achieved. After determining this instance, the strain values of the point in the location of imminent fracture (point A) are extracted at that instance, these being the critical strain points at the onset of necking. This methodology is summarized in Figure 3.

#### 2.2.2. Formability Limits by Fracture

**Figure 4:**
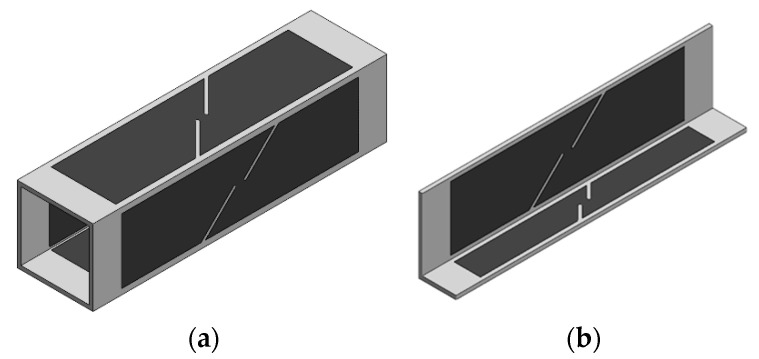
Shear specimens retrieved from (**a**) the square tube and from (**b**) the profile.

The specimens for the tensile and shear tests were extracted from the original geometries through wire electrical discharge machining. Regarding the tensile tests the specimens cut from both geometries were precisely equal (Figure 1). However, regarding the shear specimens, which have a greater width than the tensile specimens, it was not possible to extract specimens with the width recommended by the standard [22] due to size constraints. To address this, a geometric adaptation was made reducing the specimen width while maintaining the notch width and the distance between the notches. The specimens extracted from the profile and from the tube are shown in Figure 4.

The setup shown in Figure 2 enabled strain measurements during both tensile and shear tests, which were necessary to characterize the formability limits by fracture due to mode I and mode II, respectively. However, the surface strains obtained by the DIC near the fracture cannot be considered the fracture strains due to the localization and subsequent inhomogeneous deformation in the vicinity of the crack. Therefore, the procedure to determine the formability limits by fracture cannot be solely based on the DIC system but also requires some additional measurements of the specimen’s crack thickness to determine the thickness strain at fracture. These crack thicknesses measurements, $t_{F}$, were performed using a stereomicroscope Mitutoyo TM-505B (Kawasaki, Japan) with a magnification of 30×, and the thickness strain at fracture, $\varepsilon_{3F}$, was calculated by:

(2) $$\varepsilon_{3F}=ln\left(\frac{t_{F}}{t_{0}}\right),$$

where $t_{0}$ is the initial thickness; $t_{F}=\frac{1}{n}\;\sum_{i=0}^{n}t_{F}^{i}$ is the average thickness at the centre of the fracture surface of the specimen; and *n* is the number of sections measured in the specimen, which varied considerably depending on whether the measurements were performed on a shear or a tensile specimen.

The minor strain $\varepsilon_{2F}$ was assumed to remain constant after the last DIC measurement at the point for which fracture occurred, and the major strain $\varepsilon_{1F}$ is obtained by incompressibility:

(3) $$\varepsilon_{1F}=-(\varepsilon_{2F}+\varepsilon_{3F})$$

With these values, the critical strains that represent the formability limits by fracture can then be represented in the principal strain space [24].

## 3. Results

The limits analyzed in this investigation were necking and fracture, linked to two primary modes of crack development: crack opening due to tensile stresses (FFL) and crack formation resulting from in-plane shear stresses (SFFL). These three limits will be analyzed separately.

### 3.1. Necking

**Figure 5:**
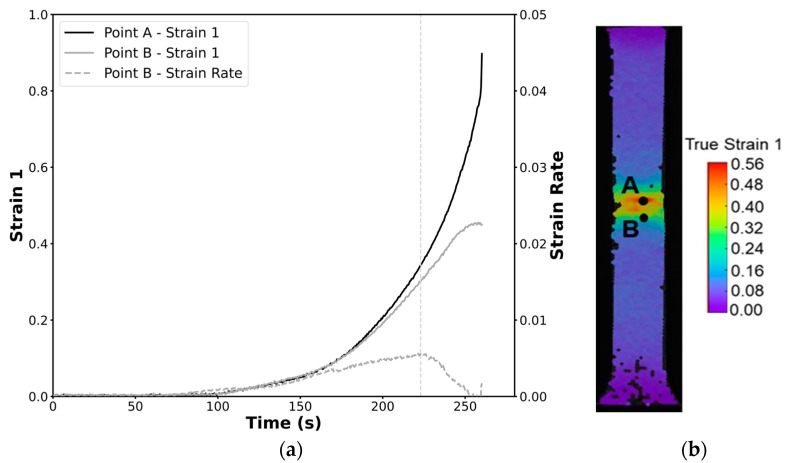
Time-dependent methodology applied to a tensile test in the longitudinal direction in a profile specimen. (**a**) Experimental time evolution of the major strains of points A and B. (**b**) Defined points A (imminent fracture) and B (boundary between the highly deformed zone and the unaffected area) in a specimen where the major strain at the onset of necking is displayed.

To determine the formability limits by necking, the time-dependent methodology, as comprehensively explained in Section 2.2.1, was applied [23]. This method requires identifying points A and B, where point A corresponds to a point in the fracture zone, and point B marks the boundary between the necking zone and the uniform deformation zone. A representative example of these points, along with the distribution of major strain over time extracted from the DIC system, is illustrated in Figure 5. This example is based on data retrieved from a tensile specimen of the profile.

**Figure 6:**
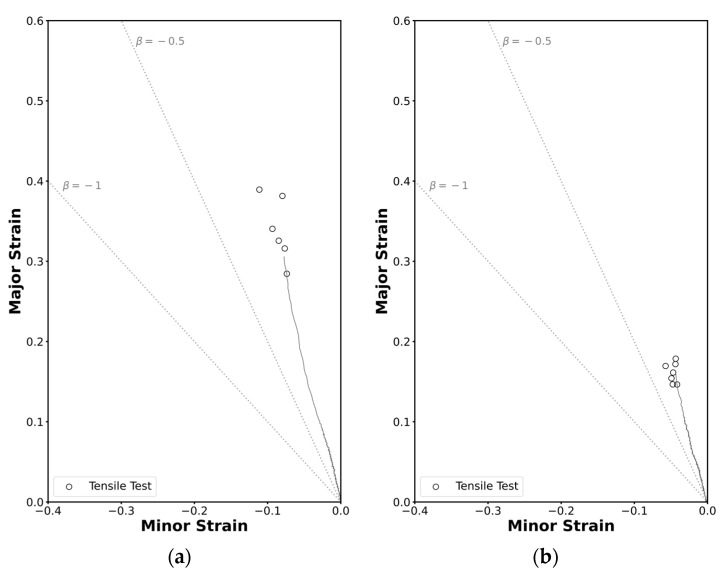
Experimental strain loading paths and formability limits of the AA6063-T6 aluminum by necking in principal strain space for the (**a**) the square tube and for (**b**) the L-section profile.

Given the consistency of results across both geometry specimens, the methodology was successfully applied. Thus, the critical strain pairs at the onset of necking for thin-walled square tubes and the profile are represented in Figure 6, alongside the corresponding strain loading paths in grey. The ratio β, defined as the ratio between the minor and major principal strains $\left(\beta =\varepsilon_{2}/\varepsilon_{1}\right),\;$ represents the slope of a proportional strain path. The lines corresponding to the strain loading paths for pure uniaxial tension (β=−0.5) and pure shear mode (β=−1) are represented as dashed lines.

Analyzing the strain loading paths, it can be observed that in both cases the slopes correspond to β≈−0.3 rather than the expected β=−0.5 for a pure uniaxial tensile state. This deviation may be attributed to the material’s anisotropy coefficient, which was inferior to 0.5, indicating that the material tends to deform more in the thickness direction than in the width direction.

### 3.2. Fracture in Mode I

**Figure 7:**
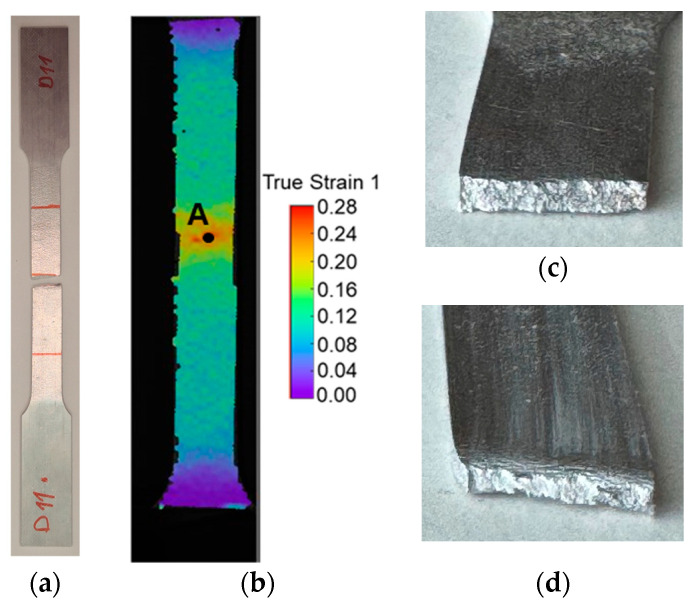
(**a**) Experimental fractured tensile specimen in the longitudinal direction and (**b**) representation of the localization of the point A in the DIC system with strain 1 distribution in the specimen. (**c**) Photograph of the fracture surface of a square tube tensile specimen. (**d**) Photograph of the fracture surface of a profile tensile specimen.

The fracture limit by tensile stresses was proposed to be determined by tensile tests in the longitudinal direction. Both the square tube and the profile were tested under the same conditions, and the results within each geometry were similar, with the repeatability assured. One typical fractured specimen and the point (A) used to extract the strain loading paths and the critical strain 2 from the DIC system can be seen in Figure 7. A photograph of the fracture surfaces of both geometry specimens is shown in Figure 7, where deformation in the profile specimen is visible.

**Figure 8:**
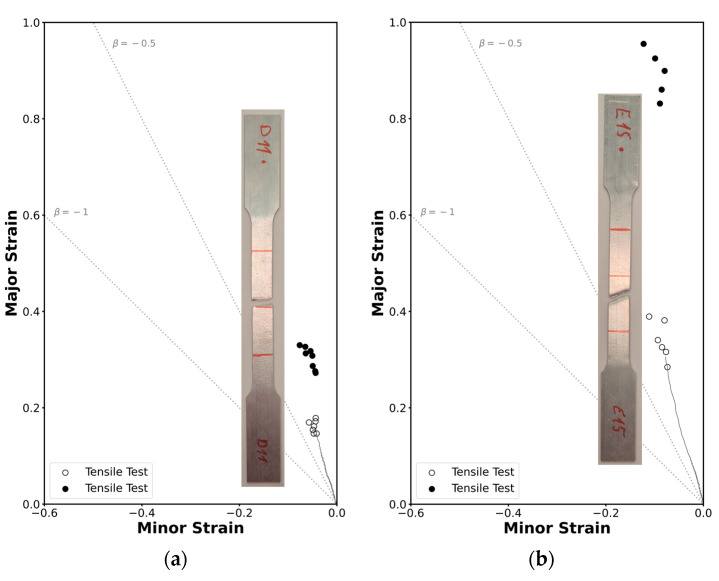
Experimental strain loading paths and formability limits of the AA6063-T6 aluminum by fracture due to tensile stresses in principal strain space for (**a**) the square tube and for (**b**) the L-section profile. Open markers represent necking points, and solid markers represent fracture points.

A representative strain loading path and the fracture strains for each specimen are represented in Figure 7, for the square tube (Figure 8a) and the profile (Figure 8b). The results were consistent among all the specimens, so it can be concluded that the methodology was successfully applied to the tensile tests.

After necking, it is possible to see that the fracture occurs after an almost plane strain deformation, as expected. It is also visible that the deformation after necking until fracture is greater in the profiles, which was also visible in the fractured specimens.

### 3.3. Fracture in Mode II

**Figure 9:**
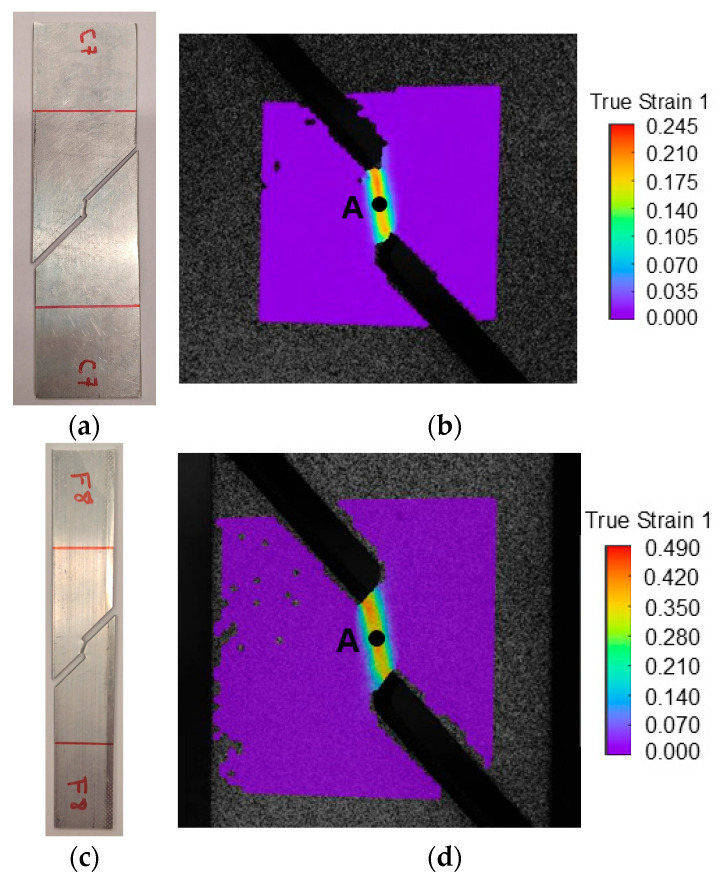
(**a**) Experimental fractured shear standard size specimens. (**b**) Representation of the location of the point A (imminent fracture) in the DIC system with strain 1 distribution in the standard size specimen. (**c**) Experimental fractured shear subsize specimens. (**d**) Representation of the location of the point A (imminent fracture) in the DIC system with strain 1 distribution in the subsize specimen.

The in-plane shear fracture forming line was determined by means of shear tests performed in the longitudinal direction of both geometries. Figure 9 shows typical fractured specimens and the point chosen to analyze the strain loading paths and subsequently extract the critical strain 1 in the last step of the DIC system.

**Figure 10:**
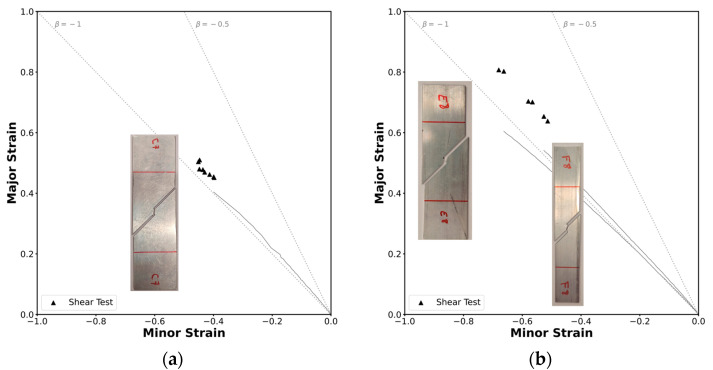
Experimental strain loading paths and formability limits of the AA6063-T6 aluminum by fracture due to in-plane shear stresses in principal strain space for (**a**) the square tube and for (**b**) the L-section profile.

The limits determined using the methodology presented previously resulted in the critical strain pairs shown in Figure 10. The strain loading paths in both geometries follow a slope that corresponds roughly to β=−1, which represents a near pure shear stress state, as expected for this test.

The critical strains at fracture in the tube are tightly grouped; however, in the profile, the results happened to be more spread out. Figure 10b shows two distinct strain loading paths: one with β<−1, corresponding to the standard specimens, and one with β>−1, associated with the narrower specimen. This difference might stem from the use of different specimen geometries, necessitated by the size constrains of the profile, as explained in Section 2.2.2. Nevertheless, the results were consistent across the specimens, allowing the conclusion that the methodology was successfully applied to the tensile tests.

### 3.4. Formability Limits Diagram

The methodology employed in this study enabled the construction of strain loading paths using data from the DIC system, the determination of critical strains at necking through a time-dependent approach, and the identification of critical strains at fracture via thickness measurements at the crack using a stereomicroscope. This comprehensive approach resulted in the determination of the formability limits by necking and fracture of this material in the square tube and in the profile. While each formability limit was discussed individually in the principal strain space, combining these limits into a single forming limit diagram provides a more comprehensive understanding of the structure’s behaviour across different deformation modes.

The formability limits by necking and by fracture due to tensile stresses and due to in-plane shear stress in the principal strain space for both geometries are represented in Figure 10. The limit curves were drawn considering an average of the points found and a theoretical slope: ‘−1’ for the FFL and ‘−1’ for the SFFL [21], resulting in four different curve equations, these being for the FFL, $\varepsilon_{1}=-\varepsilon_{2}+0.2494$ and $\varepsilon_{1}=-\varepsilon_{2}+0.7950$ for the square tube and the profile, respectively, and for the SFFL, $\varepsilon_{1}=\varepsilon_{2}+0.9118$ and $\varepsilon_{1}=\varepsilon_{2}+1.2421$ again for the square tube and the profile, respectively.

**Figure 11:**
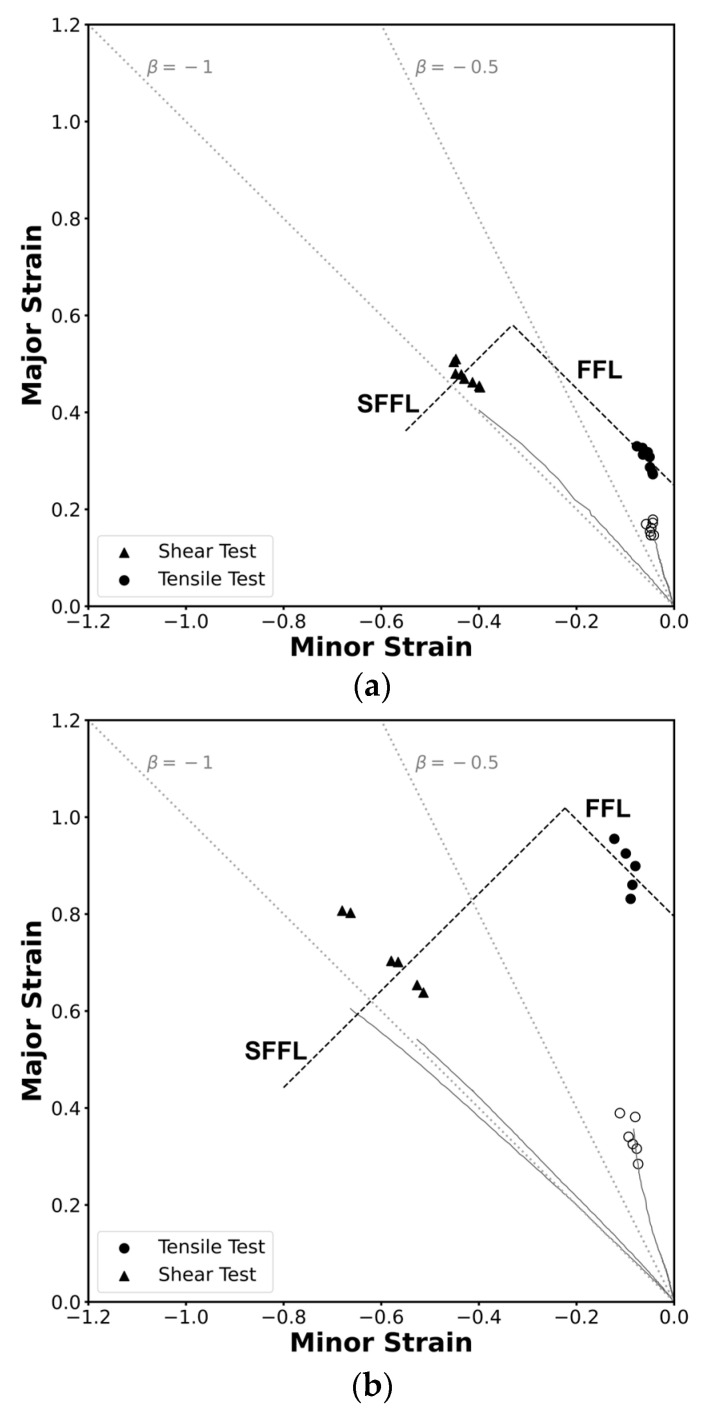
Experimental strain loading paths and formability limits of the AA6063-T6 aluminum in the principal strain space for (**a**) the square tube and for (**b**) the L-section profile. Open markers represent necking points, and solid markers represent fracture points.

In Figure 11, open markers represent the strain pairs at the onset of necking, identified using a time-dependent methodology based on DIC data. In contrast, solid markers indicate the FFL and SFFL points, corresponding to the strain values at fracture. These were determined through thickness measurements in the regions near the cracks; in these measurements it was possible to see a clear reduction in thickness for the tensile specimens, and no significant reductiont on the shear specimens.

The results obtained are similar to those observed in sheet metal forming, indicating that formability by fracture in thin-walled tube forming is constrained by the fracture forming limit (FFL) line, the shear fracture forming limit (SFFL) line, and the transition region between them within the principal strain space.

A brief comparison between the profile and the square tube reveals that the critical strain values at fracture are always higher in the profile, which indicates a greater formability capacity for this geometry under tested conditions. These differences arise despite the material being nominally the same due to variations between batches and the extrusion manufacturing process, as mentioned in the material characterization section.

## 4. Conclusions

The combination of the DIC system, the time-dependent approach, and the thickness-at-crack measurement methodology proved effective in determining the critical strains that characterize formability limits by necking and fracture in the newly studied geometries—the square tube and the profile—across tensile and double-notched shear tests. This marks the first successful application of these methodologies to thin-walled tubes with square and L-section profiles.

Anisotropy might influence the strain loading paths observed in each test, and the difference in shear specimen widths for the profile could also affect the results and must be taken into account in future investigations.

The results indicate that the thin-walled L-section profile exhibits a higher formability capacity under tensile and shear conditions compared to the thin-walled tube with square cross-section.

Future work should extend these methodologies to other tests in order to obtain more critical strain points, enabling a more comprehensive representation of the experimental FLC, FFL, and SFFL for the aluminum 6063-T6 thin-walled tube with a square cross-section and the profile with a L-section.

## Figures and Tables

**Table 1:** Geometry of the square tube and profile.

Geometry	$w_{0}$	$s_{0}$	$t_{0}$
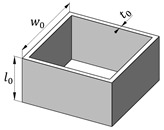	39.9	-	1.9
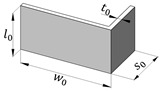	40.3	20.1	2.1

**Table 2:** Mechanical properties of the material for the square tube and the profile.

Geometry	$\sigma_{0.2\%}$ (MPa)	$\sigma_{UTS}$ (MPa)	*A* (%)	r	K (MPa)	n
Square Tube	229.9	258.2	12.7	0.489	334.1	0.0744
Profile	202.3	224.3	13.4	0.391	289.5	0.0721

## Data Availability

The original contributions presented in the study are included in the article, further inquiries can be directed to the corresponding author.

## Abbreviations

The following abbreviations are used in this manuscript:

FLC	Forming Limit Curve
FFL	Fracture Forming Limit
SFFL	Shear Fracture Forming Limit
DIC	Digital Image Correlation
ASTM	American Society for Testing and Materials
